# A Machine-Learning-Based Robust Classification Method for PV Panel Faults

**DOI:** 10.3390/s22218515

**Published:** 2022-11-04

**Authors:** Sufyan Ali Memon, Qaiser Javed, Wan-Gu Kim, Zahid Mahmood, Uzair Khan, Mohsin Shahzad

**Affiliations:** 1 Department of Defense Systems Engineering, Sejong University, Seoul 05006, Korea; 2 Department of Electrical and Computer Engineering, COMSATS University Islamabad, Abbottabad Campus, Abbottabad 22060, Pakistan

**Keywords:** convolutional neural networks, fault detection, photovoltaic cell

## Abstract

Renewable energy resources have gained considerable attention in recent years due to their efficiency and economic benefits. Their proportion of total energy use continues to grow over time. Photovoltaic (PV) cell and wind energy generation are the least-expensive new energy sources in most countries. Renewable energy technologies significantly contribute to climate mitigation and provide economic benefits. Apart from these advantages, renewable energy sources, particularly solar energy, have drawbacks, for instance restricted energy supply, reliance on weather conditions, and being affected by several kinds of faults, which cause a high power loss. Usually, the local PV plants are small in size, and it is easy to trace any fault and defect; however, there are many PV cells in the grid-connected PV system where it is difficult to find a fault. Keeping in view the aforedescribed facts, this paper presents an intelligent model to detect faults in the PV panels. The proposed model utilizes the Convolutional Neural Network (CNN), which is trained on historic data. The dataset was preprocessed before being fed to the CNN. The dataset contained different parameters, such as current, voltage, temperature, and irradiance, for five different classes. The simulation results showed that the proposed CNN model achieved a training accuracy of 97.64% and a testing accuracy of 95.20%, which are much better than the previous research performed on this dataset.

## 1. Introduction

Renewable energy, also known as green energy, comes from natural resources or processes that are constantly replaced; hence, it has less environmental impact compared to fossil fuels. Energy sources have shifted from conventional to renewable in the previous several decades, creating a noticeable paradigm change in power systems. The most common way of using solar energy is from solar panels using the photovoltaic (PV) effect to provide useable electricity [1]. According to the International Renewable Energy Agency (IRENA), during the period from 2010 and 2019, the production of renewable energy grew from 1227 GW to 2537 GW, which resulted in a an impressive 106.78% rise [2].

Similarly, PV’s power generation has expanded from 40 GW to 580 GW, a phenomenal 1350% increase in capacity from 2010 to 2019 [2]. One of the major advantage of solar energy is its easy accessibility. It is renewable and leaves almost no carbon footprint. Despite the free availability and other attractive qualities, PV systems also face challenges, such as reliability, high initial cost, fault sensitivity, and uncertainty [3]. Energy from renewable sources results in overstressed power transmission networks with compromised power quality [4], a poor voltage profile [5], and increased losses [6,7] due to the fact that these are usually connected to medium- or low-power networks. Similarly, physical, environmental, or electrical circumstances can cause faults in a PV system [8,9].

**Figure 1:**
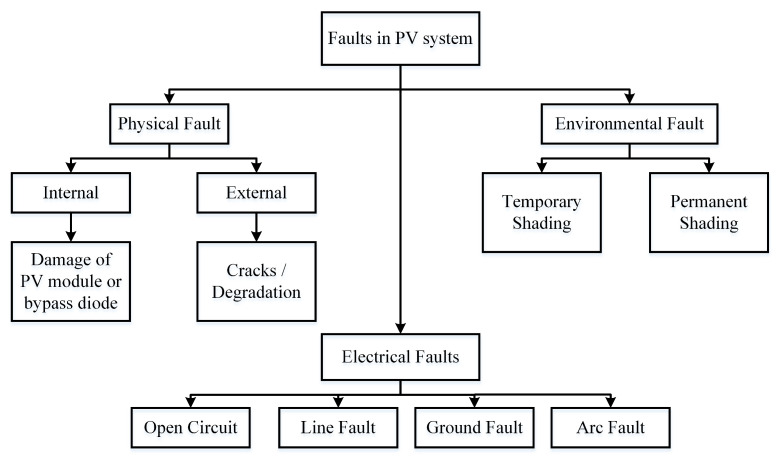
Classification of PV array faults.

Figure 1 shows that there are three major faults, which are (i) physical faults, (ii) electrical faults, and (iii) environmental faults. Obviously, the harsh outside environment in which the PV systems are installed makes them sensitive to failures and abnormalities, such as faulty wiring, Open-Circuit (OC) fault, Line-to-Line (LL) fault, Ground Fault (GF), hot spots, dust and snow accumulation, and other environmental impacts. Ultimately, PV arrays have suffered immense failures due to the aforedescribed faults, which result in decreased efficiency and a shorter lifespan [10,11]. According to the findings reported in [12], about an 18.9% reduction has been noticed in power generation due to such faults.

To overcome the power loss due to fault occurrence, the National Electric Code (NEC) suggests the usage of Ground Fault Protection Devices (GFPDs), Over-Current Protection Devices (OCPDs), and Arc Fault Circuit Interrupters (AFCIs) to detect LL, LG, and arc faults, respectively. However, the Bakersfield Fire incident in 2009 and that of Mount Holly in 2011 indicate that these devices are unable to identify the error in these specific circumstances [13]. In particular, the nonlinear properties of PV arrays, low irradiance, failure impedance, degradation, and existence of blocking diodes prevent protective devices from tripping under specific situations [13]. Therefore, faults may remain unrecognized for extended periods, demonstrating the limits of standard protection mechanisms in PV arrays. Recent studies, for instance [9,14,15], illustrated the incompatibility of such devices and conventional fault detection techniques. Based on the brief discussion so far, it is evident that fault detection and classification comprise a challenging task with very interesting contributions so far. Therefore, we believe that our work is a novel addition to this domain. The main contributions of this paper are listed below:

- We present a Convolutional-Neural-Network (CNN)-based automatic fault detection and classification method. The proposed machine learning model efficiently reduces power losses in solar PV systems by classifying faults due to its higher accuracy compared to those previously applied.
- We developed an intelligent and robust fault detection and classification technique, which is primarily based on machine learning methods. By using this novel approach, we obtained an increase in fault detection accuracy up to 97.5% from 92.64% compared to the Artificial-Neural-Network (ANN)-based model.
- To the best of our knowledge, the proposed CNN-based model has never been applied to such a big and unbalanced dataset before, for solar system fault detection application. We are optimistic that the proposed method will be a guideline for beginners and researchers who intend to initiate research in the PV domain using machine-learning-based methods.

The rest of this paper is organized as follows. Section 2 briefly presents recent advancements in fault detection in the PV domain. Section 3 familiarizes the readers with the proposed method. Section 4 presents the simulation results in detail along with the observations and discussions. Finally, Section 5 concludes the paper and provides the possible future work. In each section and to ease the readers’ understanding, the abbreviation list shows the common symbols that are used frequently in this paper and their meanings.

## 2. Related Work

**Figure 2:**
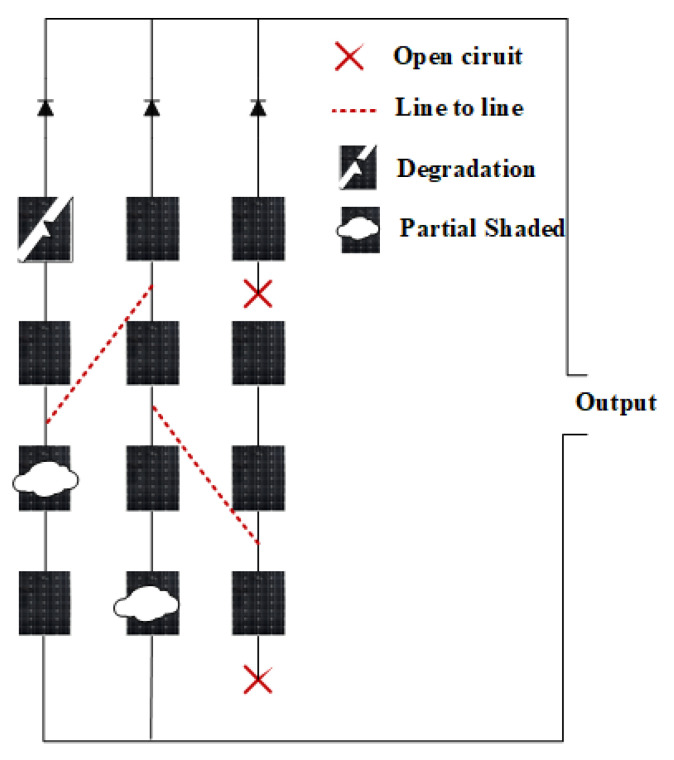
Schematic structure of different faults.

Due to numerous system irregularities, PV systems are frequently sensitive to a range of faults. These irregularities may be temporary or permanent, which ultimately lead to a degradation in system performance. Our study indicates that the Ground Faults (GFs), Short-Circuits (SCs), Open-Circuits (OCs), and shadowing are commonly occurring faults in a PV system. However, this paper considers four major and frequently occurring faults in the PV system, which are the OC, the SC, partial shading, and degradation. The schematic structure of these faults is shown in Figure 2. Below, we briefly discuss these faults:

- **The OC faults:** An OC fault in PV arrays is a disconnection issue inside a string or between two nearby strings [16]. It can occur for a variety of reasons, including a broken cable connecting two strings, an object falling on the panels, or a weak contact between two points [16].
- **The SC faults:** SC faults are caused by an accidental connection between two PV array points with potential variable values. It can occur within the same string or between two adjacent ones [17].
- **Partial shading:** Partial shading is the situation where the PV modules are partially shaded, not receiving proper sunlight to produce energy. It is a temporary situation that causes a decrease in output power [15]. There are two categories of shading [18]. The first is static shading, whereas the second is dynamic shading. Static shading is created by the accumulation of dust, leaves, and bird droppings on the glass, whereas dynamic shading is caused by a momentary shadow cast by surrounding buildings or trees.
- **Degradation:** PV systems are susceptible to degradation owing to front surface soiling, optical degradation due to continuous exposition to UV light, a rise in series resistance or a decrease in shunt resistance, a reduction in the SC current, etc. [19,20]. This error may be module-specific or occur over the entire array over time, resulting in a decline in system performance.

In [13], a fault detection model using the neural network was proposed for PV cells to detect various faults, such as the LL, the OC fault, and partial shading. The authors reported a 73.53% fault detection accuracy. In [21], a novel approach to detect microcracks using short- and long-term deep features and the Deep Convolutional Neural Network (DCNN) was developed. This method is an encouraging solution to analyze faults that appear on solar cell surfaces. In [22], the authors created a fused multi-channel CNN to identify solar cell surface imperfections. In this work, infrared and the neural networks were used to locate and construct an autonomous and robust fault detection system. In [23], faults frequently occurring in the PV domain were investigated using the CNN. To identify and characterize problems in the P modules, such as dust, shadows, and breakage, the CNN and RGB pictures were employed in [24]. This work primarily integrated the RGB images with the CNN-based model to yield encouraging results. Thermal pictures of the PV modules were classified using the SVM-based model into three categories, which were healthy, defective, and hotspot [25]. The works presented in [21,22,23,24,25] are nice efforts to detect and classify various faults in different environments and weather condition. However, few of these works employed image-processing-based methods. The pixelwise operations to detect and classify various faults consume relatively more time than a few of the methods compared therein. Moreover, image processing methods pose a major threat to developing a real-time fault detection system.

In [26], the researchers developed an SVM-based method to classify the OC and the LL faults, whereas [27,28] investigated the classification of LL faults only. These models reported good accuracy; however, the SVM can be used for binary classification and on simple datasets only. Moreover, the results of the SVM were not as effective on heavy multiclass datasets.

Recently, a few researchers developed the ANN-based fault detection model for various environments [29,30]. In particular, the accuracy of [29] was 92.64%, which is insufficient for a sensitive field. Reference [30] utilized a fairly simple dataset, which can be quickly categorized by simple classification algorithms such as the SVM and decision trees. In [31], a probabilistic-neural-network-based monitoring system was developed to detect the LL and OC faults in a 1.8 kW PV system. In their work, the I-V curve was used as an approach to distinguish among various faults. Their results were encouraging, and the probabilistic based method was one of the best performers for the ANN in the numerical classification technique. In [32], the authors also used the probabilistic method to detect the OC, the SC, the GF, and the hotspots. In [33], the developed model was designed for two different cases of faults, which were (i) single fault occurrence, which included the OC, the LL, and the shading, and (ii) multi-fault occurrence at the same time. The model was tested on a 1.22 kW PV array at the university of NCEPU, China. In [34], twelve different cases of fault were classified through the PNN, on a heavy dataset, which was collected from a 10 kW PV plant and utilized for training and testing. The authors reported a fair accuracy of 92.48% fault detection.

## 3. Proposed Method

**Figure 3:**
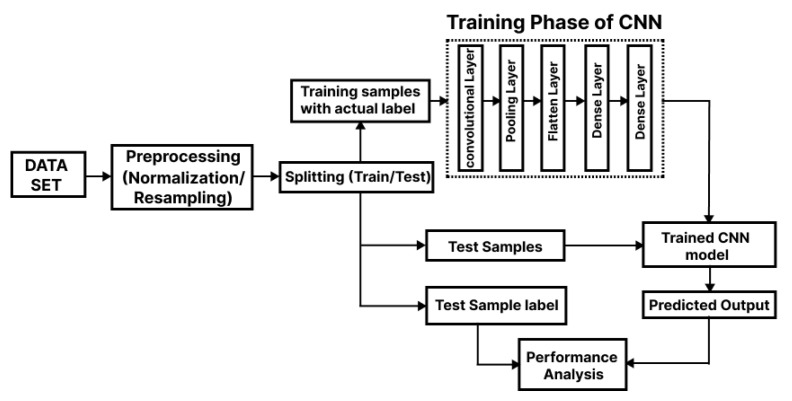
Proposed system architecture for fault diagnosis.

This section describes in detail our proposed fault detection and classification method. Numerous approaches for data normalization, training, validation, and testing of neural networks have been presented by researchers. Figure 3 shows the flowchart of the proposed methodology. While developing our algorithm, we utilized the concepts from the CNN domain.

From the literature review, we observed that the CNN is one of the most-popular ANN architectures. The CNN is specifically used in image classification, object detection, and various other computer vision and machine learning tasks. Moreover, the CNN is also extensively used in various other domains, such as Natural Language Processing (NLP) and recommender systems [35,36]. Since we intend to automate the fault detection and classification phenomenon, one of the main reasons for us to use the CNN is that it automatically extracts useful features from the input data without any manual intervention [37].

**Figure 4:**
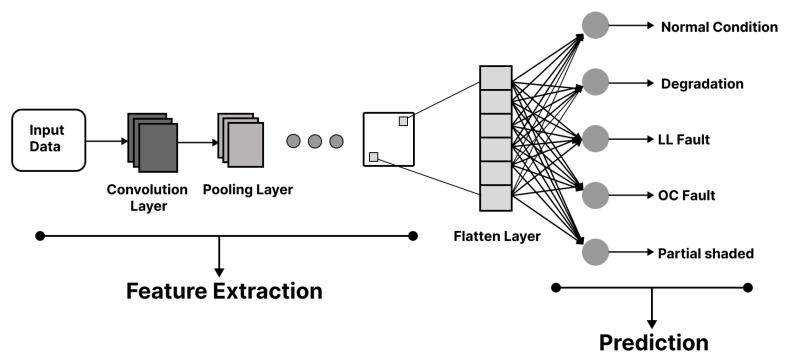
The CNN architecture.

As shown in Figure 3, the test dataset was initially preprocessed by through normalization and resampling procedures, followed by a splitting phase. In the later stage, both the test samples and test samples of the labeled faults are fed to the trained CNN module, which yields the fault classification prediction. It is worth mentioning here that the CNN architecture shown in Figure 4 used in our fault detection method comprises different layers, which include three major layers, which are the convolutional layer, pooling layer, and fully connected layer.

As shown in Figure 4, the convolutional layer is just the multiplication and accumulation process of our input signal $x=[x_{0},x_{1},x_{2},x_{3},\cdots \cdots x_{n}]$ and the kernel $k=[k_{-p},$$k_{-p+1},\cdots \cdots k_{0},\cdots \cdots k_{p-1},k_{p}]$. The kernel is slid over the whole input to execute the convolutional operation, which generates a feature map. The convolutional operation used in our can be expressed using Equation (1).

(1) $$f_{i}=\sum_{j=-p}^{p}x_{i-j}k_{j}$$

where $f_{i}$ is the feature extracted from the input signal *x*.

To learn some useful nonlinear patterns from the input, a nonlinear activation function was applied to the model. Different activation functions, such as sigmoid and tanh, are also used. However, the most commonly used is the Rectified Linear Unit (ReLU), which is expressed by Equation (2).

(2) $$y=max(0,x_{i})$$

As shown in Figure 4, after convolution, pooling layers are added, which help reduce the spatial dimension of the representation to minimize the computations. This also helps to reduce the number of parameters, which in later stages helps with training time reduction. Normally, two functions are used in the pooling operation, which are average pooling and max pooling. In our model, we used max pooling. The outputs of the pooling layers are fed to the Fully Connected (FC) layer, which is the mandatory layer of the ANN. In the CNN, the convolutional layer and pooling layer extract the features from the data, whereas the FC layer performs the classification.

The SoftMax function is used as the activation function for the final classification in the output layer and computes the probability values of all the classes using Equation (3).

(3) $$P{\left(\vec{Z}\right)}_{i}=\frac{e^{Z_{i}}}{\sum_{j=1}^{J}e^{Z_{i}}}$$

where $Z_{i}$ is the input vector of the SoftMax function. Different range $P{\left(\vec{Z}\right)}_{i}$ values were selected according to the number of classes.

Algorithm 1 illustrates the CNN model designed for the classification of fault occurrence in the PV panel. As shown in lines (2)–(3) of Algorithm 1, firstly, the dataset is preprocessed through normalization and resampling, then it passes through a convolution filter of size 6 × 1, which extracts the useful features, and that feature extraction results in the feature map. The extracted feature *F* is then passed through the MaxPool filter of size 2 × 1 shown in line (4) of Algorithm 1. Further, as shown in line (5) of Algorithm 1, the output of the pooling layer is converted into a vertical vector by the flatten layer. The aforementioned data are now processed by the fully connected layer as shown in line (6), where advanced features and the probabilities of all classes are computed through the dense layers using 16 nodes. Finally, as shown in line (7), the output layer with 5 nodes is applied. The 5 nodes, which yield the classification, are normal condition, degradation, the LL faults, the OC faults, and partial shading.
**Algorithm 1:** CNN algorithm for PV fault detection.**Input:** [X,y]; label raw data.**Preprocessing:** normalization and resampling**Convolution layer:** CL←[X,y] raw data sent to the CNN for feature extractionF: feature vector (feature map) extracted from convolution layer**Pooling layer:** MaxPool←F;[X,y] downsampling feature map with MaxPool $F_{D}$; downsampled feature vector or feature map**Flatten layer:**$F_{L}$←$F_{D}$; change dimension of $F_{D}$$F_{L}$: flatten feature map**Fully connected layer:**$F_{C}$←$F_{L}$; compute more advanced features and give probability values of each classP: probabilities of all the classes**Output:** classifies the test data

Now that the algorithm has been developed, in the next section, we discuss in detail the simulation results along with our findings.

## 4. Simulation Results

We performed detailed simulations using the Google Colab platform, which provides significant and substantial resources in the form of the Google Compute Engine with 12 GB of RAM and 107 GB of disk storage. Before we proceed further, below, we briefly describe the datasets that were used in our work.

### 4.1. Dataset Description

**Dataset-1:** This dataset was developed by Lazzaretti et al. [29] in 2020. This dataset contains different features, such as current, voltage, temperature, and irradiation level. The dataset contains these features for five distinct conditions, which are the OC, the LL, partial shading, degradation, and normal condition. In addition, in our work, we selected the following six PV features as the input to train the model:

- $x_{1}:$ voltage of string 1;
- $x_{2}:$ voltage of string 2;
- $x_{3}:$ current of string 1;
- $x_{4}:$ current of string 2;
- $x_{5}:$ irradiation level;
- $x_{6}:$ temperature of PV panel;
- $x_{7}:$ fault label.

**Figure 5:**
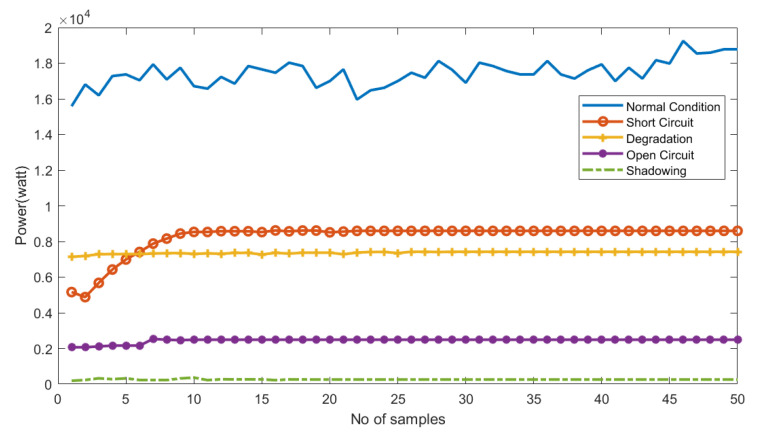
The PV power production under normal and faulty conditions.

Figure 5 shows the comparisons of the power produced during a normal condition and four other faulty conditions. It is obvious from the green line in Figure 5 that the power produced under normal circumstances is much greater than that under faulty conditions. In the case of shadowing, the PV panel produces the least current. As a result, the power produced is less than 10 Watts. For other faults, such as short-circuit, degradation, and open-circuit faults, as shown in Figure 5, the amount of produced power is well below the desired level.

### 4.2. Dataset Preprocessing

In our work, the dataset was preprocessed before being fed to the CNN module. To accelerate the learning procedure and ensure fast convergence, the dataset was initially normalized using standard normalization techniques, as shown by Equation (4).

(4) $$z=\frac{x-u}{s}$$

where *z* is the normalized value replacement for *x*, *u* is the sample’s mean, and *s* is the standard deviation of each feature. After normalization, oversampling was performed as the dataset was imbalanced, because the normal condition contained 886,884 samples, whereas the SC class contained only 1228 samples [38]. Later, the dataset was randomly partitioned into two subsets, which were the training dataset (80%) and testing dataset (20%).

### 4.3. The CNN Performance Analysis

To analyze the performance of a neural network or any other machine learning model, learning curves are the most widely used tools. A learning curve is the plot of the model performance based on experience. Looking at the learning curves, one can easily say how well the model finds a relation between the input and output, as well as whether the model is overfit, underfit, or the best-fit model. Usually, we can find two different learning curves.

**Training curves:** These curves indicate an estimation of how well the model is learning over time from the training dataset.

**Validation curves:** These curves indicate an estimation of how well the model is generalizing based on how it behaves for unseen data.

The purpose of training a machine learning model is to find the best-fit model.

**Figure 6:**
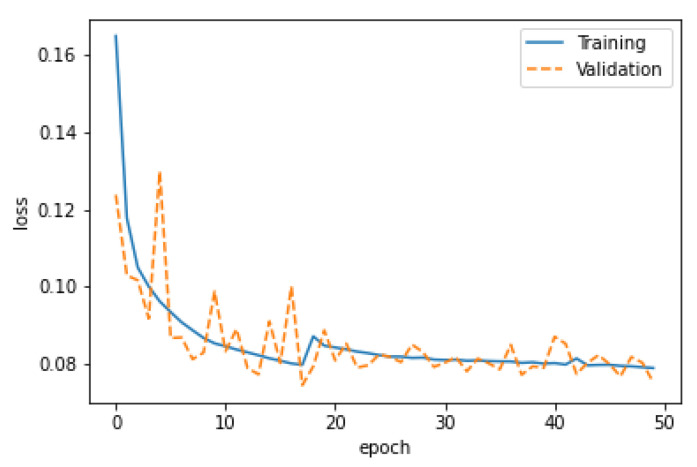
Best-fit model.

The best-fit model is identified by the validation and training losses, which decrease the stability point along with a minimal space between the two last loss values. A typical loss function is cross-entropy. The loss function in our model is defined by Equation (5).

(5) $$loss=-\frac{1}{N}\sum_{i=1}^{N}y_{i}log\hat{y_{i}}+\left(1-y_{i}\right)log\left(1-\hat{y_{i}}\right)$$

where *N* is the total number of examples, $y_{i}$ is the actual value, and $\hat{y_{i}}$ is the predicted value. The losses’ learning curves for the dataset are shown in Figure 6. Clearly, it can be seen in Figure 6 that, for the epochs set to 50, both the training and validation curves fit best in the given range.

### 4.4. Accuracy Curves

**Figure 7:**
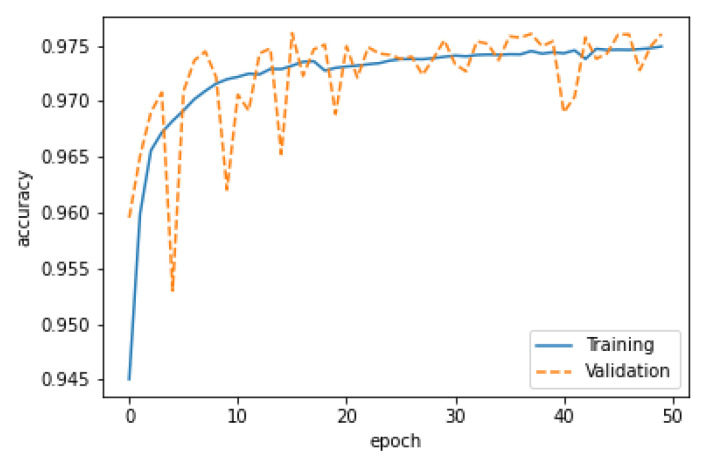
Training and validation accuracy.

The accuracy parameter is frequently used to evaluate a model’s performance. It is defined as the ratio of correct predictions to all predictions. In our work, the accuracy of a model is calculated by using Equation (6).

(6) $$Accuracy=\frac{TN+TP}{TN+FN+FP+TN}$$

where the term *TN*stands for True Negatives and indicates the number of negatively classified cases that were correctly identified. Similarly, *TP* denotes True Positives and depicts the quantity of correctly identified positive cases. The term *FP* denotes the number of False Positives that were mistakenly categorized as positive, while *FN* denotes the number of False Negatives that were mistakenly classified as negative. Figure 7 shows the training and validation accuracy of our proposed CNN-based model. It can be observed from Figure 7 that, up to 20 epochs, the validation curve tried to match the training curve. After 40 epochs, the difference between the curves was minimized, which further indicates that the model was well trained at 50 epochs. Observe that the difference between the training curves and validation curves is the basic way to find a model’s behavior. In our case, the difference was much less, which shows that the model will predict well for unseen data.

### 4.5. Confusion Matrix

**Figure 8:**
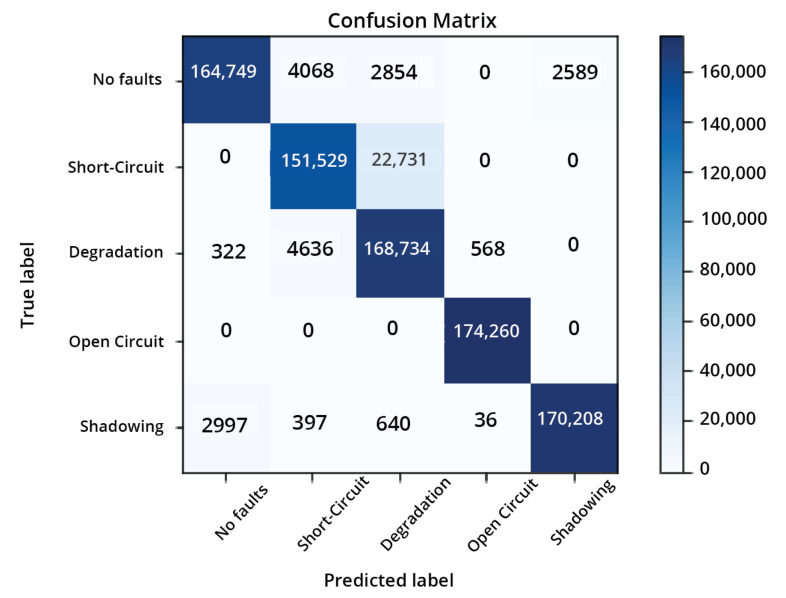
Confusion matrix.

The confusion matrix shows the summary of how a model performs on new data, which is usually the test data. The confusion matrix of our model is shown in Figure 8. In Figure 8, the x-axis shows the predictions and the y-axis shows the actual labels of the classes.

The accuracy achieved for our model for the normal condition, the SC, degradation, the OC, and shadowing was 94.54%, 86.95%, 96.82%, 100%, and 97.67%, respectively. The total testing accuracy of the model was 95.20%.

### 4.6. Comparison

From the aforementioned analysis, we observed that our model was well trained and yielded encouraging training and testing accuracy. In this section, we compare our work with four recent works. Table 1 shows the comparison of our work with other models that were recently published on fault detection in the PV domain. For a fair comparison, we also performed detailed experiments on a multi-class fault dataset, which we briefly describe in Section 4.1. A few of the important observations from Table 1 are listed below:

- It can be seen in Table 1 that, among all the compared methods, the work reported in [13] yielded the least accuracy of 73.53% to classify the multi-class faults, such as the LL, the OC, and partial shading faults. This was due to the fact that, in their method, the last few layers of a pre-trained AlexNet are fine-tuned to produce several types of outputs. Similarly, the authors also obtained features from the fully connected (fc7) layer of a pre-trained AlexNet and then used this in conjunction with classical ML methods for classification.
- The works reported in [26,32] yielded the same 97% fault detection accuracy. In particular, the developed method in [26] uses the SVM framework to classify only the LL and the OC faults, whereas Reference [32] successfully classified the GF, the OC, the SC, and hotspot faults by utilizing the PNN framework to yield a similar accuracy.
- Moreover, the authors of [29] also achieved a 92.64% fault detection accuracy for the multi-class faults, such as the OC, the SC, degradation, and shadowing. This is an interesting solution in the fault detection domain, where the authors introduced a recursive linear model to detect faults in the system, primarily through the use of irradiance on the PV panel as the input signals and power as the output.
- Similarly, the work published in [33], utilized a probabilistic framework to classify various faults and, thereby, yielded a good accuracy of 94.69%. This model is also a novel contribution to the fault detection domain and achieved encouraging results by employing several sequential steps. Initially, the authors analyzed the performance of seven indicators to accurately predict the nonlinear output behavior of the PV system under changing environmental conditions. Moreover, several fault cases, considering single-fault types and another three faults considering multiple fault types, were also investigated. In the final stages of this sequential algorithm, the typical fault types were classified and detected using sequential probabilistic neural network models, which gave an effective classification of the data inputs.
- The aforedescribed works are sound solutions in the PV domain to reliably detect and classify various faults. However, as seen in Table 1, the proposed fault detection model yielded the highest accuracy among all the compared works. Moreover, our developed CNN-based fault detection model yielded much better accuracy than [13], who also used the CNN framework. On the multi-class dataset developed by [29], we were able to improve the multi-class fault detection accuracy by 5% and 24.11% compared to [13]. Our study indicates that the higher fault detection accuracy in our work was made possible due to the intelligent utilization of the Application Program Interfaces (APIs), which encouraged us to design and operate different layers of the CNN model. Moreover, Google Tensorflow perfectly created and managed these APIs. Furthermore, our proposed method allows real-time fault detection and classification. The training samples along with the actual labels were fed to the training phase of the CNN, which involved various layers, such as convolutional, pooling, flatten, and dense layers, resulting in the intelligently trained CNN model. Later, this model handled the test samples effectively.

**Table 1:** Accuracy comparison of our model with previous work.

Ref.	Model	Faults under Consideration	Accuracy
[13]	CNN	LL, OC, partial shading	73.53%
[26]	SVM	LL, OC	97%
[29]	ANN	OC, SC, degradation, shadowing	92.64%
[33]	PNN	GF, OC, SC, hotspot	97%
**Proposed Model**	**CNN**	**OC**, **SC**, **degradation**, **shadowing**	**97.64%**

### 4.7. Computational Complexity

**Figure 9:**
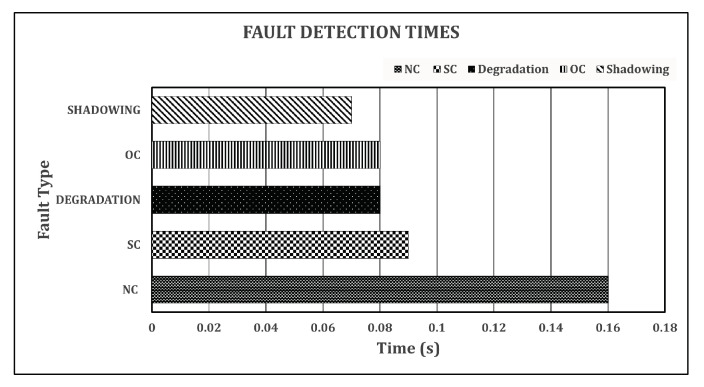
Fault detection times.

Figure 9 shows the detailed computational complexity in terms of the time consumed to detect and classify various faults. As can be seen in Figure 9, our model took a one-time training time of 58 min and 18 s for 50 epochs, and this can vary according to the number of epochs set for the simulation; it can also be affected by the RAM of the PC on which the model is trained. For fault detection, such as for the Normal Condition (NC), it consumed 0.16 s. For the SC faults, it took 0.09 s. For degradation and Open-Circuit (OC), only 0.08 s was used to detect and classify these faults. Finally, for shadowing, our proposed method took 0.07 s to detect and classify this fault.

## 5. Discussion

Although the aforedescribed analysis shed detailed light on the performance of the developed machine-learning-based fault detection method, for the readers’ more in-depth information, below, we briefly discuss the following important points:

- Our study indicated that there is a severe lack of standard protocols to generate and analyze various faults. Similarly, there is a scarcity of available diverse public datasets. We are optimistic that future studies will present more diverse datasets to detect, identify, and classify various faults.
- Our proposed fault detection model classified five different types of fault cases efficiently. However, we observed that the proposed CNN-based fault detection model is heavy compared to other machine algorithms, such as the Discrete Fourier Transform (DFT) and the SVM. Moreover, our proposed model presented a comparable performance to the other works listed in Table 1.
- Our proposed model works in a near real-time fault detection and classification manner. Similarly, our findings suggest that shadowing events are caused by real shadowing, which makes them a challenging task to be accurately characterized. Similarly, controlled shadowing normally increases the performance for a class.

## 6. Conclusions

Maintaining continuous energy production in PV systems is a critical issue for power utilities, which has attracted much attention from the academic community. Various methods are appearing every day to automate and mitigate the production deviations in PV plants. PV systems are susceptible to various faults and breakdowns. An early fault diagnosis is critical for the PV system’s effectiveness and reliability. We presented a CNN-based fault detection algorithm for PV arrays to properly distinguish failure categories. This model was trained on a heavy dataset. The dataset was preprocessed before being fed to the CNN module. The proposed model was well trained and yielded a training accuracy of 97.64%, as well as a validation accuracy of 97.67%.

In the future, we intend to modify the CNN architecture of the model to further improve the detection and classification accuracy for multiple faults’ detection. Moreover, we also aim to optimize the proposed model to be reliably used in other fields, such as fault detection in wind turbines.

## Data Availability

Data can be provided upon request.

## Abbreviations

The following abbreviations are used in this manuscript:

AFCI	Arc Fault Circuit Interrupter
ANN	Artificial Neural Network
API	Application Programming Interface
CNN	Convolutional Neural Network
DCNN	Deep Convolutional Neural Network
DT	Decision Tree
FC Layer	Fully Connected Layer
GF	Ground Fault
GFPD	Ground Fault Protection Device
GW	Gigawatt
IREA	International Renewable Energy Agency
KW	Kilowatt
LL	Line-to-Line
NEC	National Electric Code
NLP	Natural Language Processing
OC	Open-Circuit
OCPD	Over-Current Protection Device
PV	Photovoltaic
PNN	Probabilistic Neural Network
RGB	Red Green Blue
SC	Short-Circuit
SVM	Support Vector Machine
UV	Ultraviolet
